# Association of Emergency Department Payer Mix with ED Receipt of Telehealth Services: An Observational Analysis

**DOI:** 10.5811/westjem.2021.9.53014

**Published:** 2022-01-31

**Authors:** Kori S. Zachrison, Margaret E. Samuels-Kalow, Krislyn M. Boggs, Sijia Li, Emily M. Hayden, Carlos A. Camargo

**Affiliations:** * Massachusetts General Hospital, Department of Emergency Medicine, Boston, Massachusetts; † Harvard Medical School, Department of Emergency Medicine, Boston, Massachusetts

## Abstract

**Introduction:**

Telehealth is commonly used to connect emergency department (ED) patients with specialists or resources required for their care. Its infrastructure requires substantial upfront and ongoing investment from an ED or hospital and may be more difficult to implement in lower-resourced settings. Our aim was to examine for an association between ED payer mix and receipt of telehealth services.

**Methods:**

Using data from the National Emergency Department Inventory (NEDI)-USA 2016 survey, we categorized EDs based on receipt of telehealth services (yes/no). The NEDI-USA data for EDs in New York state was linked with data from state ED datasets (SEDD) and state inpatient data (SID) to determine EDs’ payer mix (percent self-pay or Medicaid). Other ED characteristics of interest were rural location, academic status, and annual ED visit volume. We compared EDs with and without telehealth receipt, and used a logistic regression model to examine the relationship between ED payer mix and telehealth receipt after accounting for other ED characteristics.

**Results:**

Of the 162 New York EDs in the SEDD-SID dataset, 160 (99%) were linked to the NEDI-USA dataset and 133 of those responded (83%) to the survey. Telehealth receipt was reported by 48 EDs (36%, 95% confidence interval [CI], 28–44%). Emergency departments with and without telehealth receipt were similar (all P >0.40) with respect to rurality (6% vs 9%, respectively), academic status (13% vs 8%), and annual volume (median 36,728 vs 43,000). By contrast, median percent of Medicaid or self-pay patients was lower in telehealth EDs (36%) vs non-telehealth EDs (45%, P = 0.02). In adjusted analysis, increasing proportion of Medicaid and self-pay patients was associated with decreased odds of telehealth receipt (odds ratio 0.87 per 5% increase; 95% CI, 0.77–0.99). Rural location, academic status, and ED volume were not significantly associated with odds of ED telehealth receipt in the adjusted model.

**Conclusion:**

Among EDs in the state of New York, increasing proportion of self-pay and Medicaid patients was associated with decreased odds of ED telehealth receipt, even after accounting for rural location, academic status, and ED volume. The findings support the need for additional infrastructural investment in EDs serving a greater proportion of disadvantaged patients to ensure equitable access.

## INTRODUCTION

As telehealth transforms the delivery of healthcare, emergency departments (ED) in particular stand to benefit. Emergency departments have varying level of resources, with some rural EDs lacking consultant availability and even physician staffing.^1^,^2^ Patients presenting to less resourced or rural EDs are often transferred to urban referral centers to access resources or specialty care. Yet where some care is becoming increasingly regionalized and concentrated in fewer centers (eg, definitive pediatric hospital care),^3^ telehealth provides an opportunity to counter this trend. By virtually bringing a consulting specialist to a patient in a rural ED, telehealth can mitigate this gap in resource availability for those sites. Rather than bringing patients to the resources, telemedicine enables a strategy of bringing the resources to the patient. Potential benefits include enabling patients to receive medical care closer to their home and, simultaneously, enabling hospitals to provide a level of resource to patients that would not have otherwise been possible. In doing so, smaller hospitals may be able to retain more patients and maintain a higher census and more favorable financial status as well.

Yet telehealth infrastructure requires substantial upfront and ongoing investment from an ED or hospital, and this may be more difficult to implement in lower-resourced settings. We have previously found that smaller, rural EDs are the least likely to receive telehealth services.^4^ These findings are concerning as the expansion of telehealth has potential to exacerbate inequities in care access when the hospitals that could most benefit from telehealth are least likely to have the resources to receive telehealth services. The objective of this study was to further explore the potential connection between level of resources and ED receipt of telehealth services, specifically to examine for an association between ED payer mix and receipt of telehealth services. We hypothesized that EDs with higher proportion of self-pay or Medicaid patients would have lower odds of receiving telehealth services, after accounting for other ED characteristics.

## METHODS

Using data from a survey of all US EDs open in 2016, as part of the National ED Inventory (NEDI-USA), we identified EDs’ receipt of telehealth services. This one-page survey was administered in 2017 to characterize EDs open in 2016. The methods, including those of telehealth status ascertainment, have been previously reported.^4^,^5^ We included all EDs that were open 24/7 and available for use by the general public, including hospital-based and freestanding EDs. There was no incentive to participate. Surveys were completed on paper, online, or by telephone. For respondents completing the survey by telephone, a standard script was used to define telehealth as needed. We categorized EDs based on receipt of telehealth services (yes/no) based on response to the survey item “Does your ED *receive* telemedicine services for patient evaluation?” The study was approved by the Massachusetts General Hospital Institutional Review Board.

The NEDI-USA data for EDs in New York State was linked with data from state ED datasets (SEDD) and state inpatient data (SID)^6^ to determine EDs’ payer mix (percent self-pay or Medicaid). Other ED character-istics of interest were rural location (based on location outside of a core-based statistical area), academic status (based on presence of an emergency medicine residency), and annual ED visit volume. We used t-tests and chi-square test to compare EDs with and without telehealth receipt. A multivariable logistic regression model examined the relationship between ED payer mix and telehealth receipt after accounting for other ED characteristics. We tested for an interaction between ED volume and proportion of payer mix to determine whether the relationship between payer mix and likelihood of telehealth use varied by volume. Because the interaction was not significant it was dropped from the model for ease of interpretation.

## RESULTS

Of the 162 New York State EDs in the SEDD-SID linked dataset, 160 (99%) were linked with the NEDI-USA dataset and 133 (83%) responded to the NEDI-USA survey (Figure). Telehealth receipt was reported by 48 EDs (36%, 95% confidence interval [CI] 28–44%). In bivariate comparisons, EDs with and without telehealth receipt were similar (all *P* >0.40) with respect to rurality (6% vs 9%, respectively), academic status (13% vs 8%), and annual ED visit volume (median 36,728 vs 43,000). By contrast, median percent of Medicaid or self-pay patients was lower in telehealth EDs vs non-telehealth EDs (36% vs 45%, respectively; *P* = 0.02).

In adjusted analysis, the results were similar. Rural location, academic status, and annual ED visit volume were not significantly associated with odds of ED telehealth receipt in the adjusted model (Table). By contrast, increasing proportion of Medicaid and self-pay patients was associated with decreased odds of telehealth receipt (odds ratio 0.87 per 5% increase, 95% CI, 0.77–0.99).

## DISCUSSION

In summary, among EDs in New York State, increasing proportion of self-pay and Medicaid patients was associated with decreased odds of ED telehealth receipt, even after accounting for rural location, academic status, and annual ED visit volume. While we are not aware of prior research specifically examining the relationship between ED payer mix and telehealth receipt, this finding is consistent with other literature demonstrating patient-level disparities in access by insurance status.^7^ Prior research has also demonstrated increased likelihood of ED closure among safety-net EDs and EDs serving a higher share of population with public insurance.^8^,^9^ It may follow that EDs serving a greater proportion of disadvantaged patients may have less telehealth access. Particularly in 2016, technological equipment and internet connectivity infrastructure were expensive, and the cost may have been prohibitive for EDs in hospitals operating with thin or even negative financial margins.^10^

Payment policy may play a role. In the context of these pre-COVID-19 pandemic data, reimbursement for telemedicine was extremely limited and mostly only available for patients in rural areas (with the exception of coverage for telestroke introduced with the FAST Act in 2017).^11^ Yet even for rural hospitals the payment structure has been a barrier. In both the commercial and academic hub-and-spoke model, EDs with telehealth typically pay subscription fees directly to the telehealth provider. There are theoretically two ways in which these sites could then recoup those costs: 1) the ED could credential all telehealth consultants at their site in order to bill professional fees on their behalf; or 2) the ED would need to successfully avoid transfer and locally admit a large proportion of patients so that the increase in locally admitted bed-days would offset the expense.^12^ However, this may not be worthwhile in the context of low volumes and high administrative burden. In addition, if those bed-days are for patients with Medicaid or self-pay, the potential financial gains of avoiding transfer may be further limited. Finally, there may be alternative reimbursement strategies that may be suitable, such as expanded allowance for physicians to be remote from patients; this strategy has been well established in diagnostic radiology.

From a clinical perspective, telehealth may be considered a worthwhile – and moreover, an important – investment, enabling higher quality care delivery for patients by providing access to resources and consultants that would not have otherwise been available. There is a successful model for telehealth implementation among rural critical access hospital EDs in the Midwest with Avera Health.^13^ However, in some of these EDs, the business case may involve the substitution of nonphysician providers or non-emergency physicians for EM-trained physicians, with availability of backup tele-emergency physicians.^14^ This transition from emergency physicians to non-physician clinicians may have alternative implications for quality of care delivery if patients no longer have access to emergency care from emergency physicians.

## LIMITATIONS

This study has limitations. Telehealth receipt was identified based on survey responses, which were not validated and were dependent on respondents’ knowledge of programs, although we aimed to mitigate this by surveying ED directors and others in leadership who are knowledgeable about ED operations. Survey responses may also be subject to social desirability bias. Our data is from a single state and may not be generalizable to other settings. Furthermore, much has changed and may be expected to continue changing in the telehealth landscape since 2016, including lower costs of technology and changes in payment policy during the coronavirus 2019 public health emergency. For example, the Centers for Medicare and Medicaid Services 1135 waiver enabled broad expansions in telehealth reimbursement improving access to virtual care.^15^,^16^

While these changes are temporary under the public health emergency declaration and the future of telehealth reimbursement policy remains unclear,^17^ it is likely that the post-pandemic reimbursement landscape will be distinct from the 2016 results presented here. Further research is warranted to confirm these findings in other settings and with more recent data. Finally, while the benefits of telemedicine on morbidity and mortality are well established in some conditions (eg, telestroke),^18^ the relative costs and benefits in other domains of care may be debated.

## CONCLUSION

These novel findings support additional infrastructural investment in EDs serving a greater proportion of disadvantaged patients to ensure equitable access, and further development of strategies to reduce costs and improve reimbursement payment models to address this disparity in access.

## Figures and Tables

**Figure:**
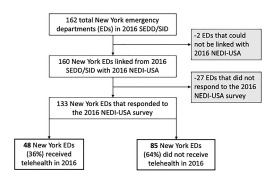
Flowchart of emergency departments included in the study. *SEDD/SID*, State Emergency Department Database/State Inpatient Database; *NEDI-USA*, National Emergency Department Inventory-USA.

**Table:** Unadjusted and adjusted odds of telehealth receipt in New York State, by emergency department characteristics.

	Unadjusted odds ratio	95% CI	Adjusted odds ratio	95% CI
Rural location	0.64	(0.16–2.54)	0.54	(0.13–2.29)
Academic ED	1.59	(0.50–5.04)	1.63	(0.39–6.89)
Annual ED volume (per 5,000 increase)	1.00	(0.97–1.03)	1.00	(0.96–1.05)
Percent Medicaid or self-pay (per 5% increase)	0.89	(0.80–0.99)	0.87	(0.77–0.99)

*CI*, confidence interval; *ED*, emergency department.
